# 2,9-Dimethyl-6*H*,13*H*-5:12,7:14-dimethano­dibenzo[*d*,*i*][1,3,6,8]tetraazecine

**DOI:** 10.1107/S1600536809038380

**Published:** 2009-09-26

**Authors:** Augusto Rivera, Mauricio Maldonado, Jaime Ríos-Motta, Diego González-Salas, Bruno Dacunha-Marinho

**Affiliations:** a Departamento de Química, Universidad Nacional de Colombia, Bogotá, AA 14490, Colombia; b Ed. CACTUS, Campus Sur, Unidade de Raios X, Universidad de Santiago de Compostela, 15782, Spain

## Abstract

In the title structure, C_18_H_20_N_4_, the aromatic rings are almost orthogonal [81.6 (2)°]. The mol­ecule has symmetry 2 since it is situated on a crystallographic twofold axis. There are only weak inter­molecular inter­actions present in the structure, notably C—H⋯π-electron ring inter­actions. The ^1^H and ^13^C NMR spectra are in accordance with the X-ray structure analysis.

## Related literature

For the synthesis of the title compound, see: Volpp (1962 ▶); Kuznetsov *et al.* (2007 ▶). For related structures, see: Dickinson & Raymond (1923 ▶); Murray-Rust (1974 ▶); Murray-Rust & Ridell (1975 ▶); Murray-Rust & Smith (1975 ▶); Glister *et al.* (2005 ▶); Rivera *et al.* (2007 ▶); Volpp (1962 ▶). For the chemical reactivity of cyclic aminals, see: Rivera *et al.* (2005 ▶); Rivera & Maldonado (2006 ▶).
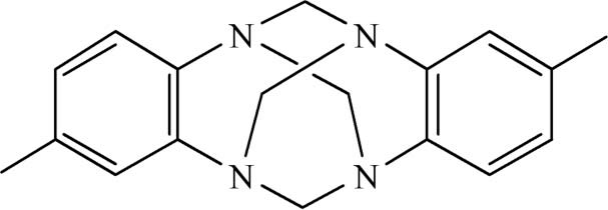


## Experimental

### 

#### Crystal data

C_18_H_20_N_4_
*M* *_r_* = 292.38
Orthorhombic, 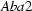

*a* = 9.9777 (3) Å
*b* = 18.8351 (4) Å
*c* = 7.6963 (2) Å
*V* = 1446.37 (7) Å^3^
*Z* = 4
Mo *K*α radiation
μ = 0.08 mm^−1^
*T* = 100 K
0.16 × 0.15 × 0.06 mm

#### Data collection

Bruker APEXII CCD diffractometer
Absorption correction: multi-scan (**SADABS**; Sheldrick, 2006 ▶) *T*_min_ = 0.872, *T*_max_ = 0.995
10245 measured reflections
807 independent reflections
737 reflections with *I* > 2σ(*I*)
*R*_int_ = 0.041

#### Refinement

*R*[*F* ^2^ > 2σ(*F* ^2^)] = 0.033
*wR*(*F* ^2^) = 0.090
*S* = 1.06
807 reflections
102 parameters
1 restraint
H-atom parameters constrained
Δρ_max_ = 0.18 e Å^−3^
Δρ_min_ = −0.18 e Å^−3^

### 

Data collection: *APEX2* (Bruker, 2005 ▶); cell refinement: *SAINT* (Bruker, 2005 ▶); data reduction: *SAINT*; program(s) used to solve structure: *SIR97* (Altomare *et al.*, 1999 ▶); program(s) used to refine structure: *SHELXL97* (Sheldrick, 2008 ▶); molecular graphics: *ORTEP-3 for Windows* (Farrugia, 1997 ▶); software used to prepare material for publication: *WinGX* (Farrugia, 1999 ▶).

## Supplementary Material

Crystal structure: contains datablocks global, I. DOI: 10.1107/S1600536809038380/fb2167sup1.cif

Structure factors: contains datablocks I. DOI: 10.1107/S1600536809038380/fb2167Isup2.hkl

Additional supplementary materials:  crystallographic information; 3D view; checkCIF report

## Figures and Tables

**Table 1:** Geometry of C—H⋯ *Cg* interactions (Å,°)

Contact	C–H	C⋯*Cg*	H⋯*Cg*	C—H⋯*Cg*
C2–H2⋯*Cg*^i^	0.95	3.509 (2)	2.68	147
C10–H10*B*⋯*Cg*^ii^	0.98	3.559 (2)	2.61	163

Symmetry codes: (i) 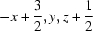; (ii) 
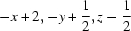
. *Cg* denotes the centroid of the benzene ring C1–C6.

**Fig. 1.**
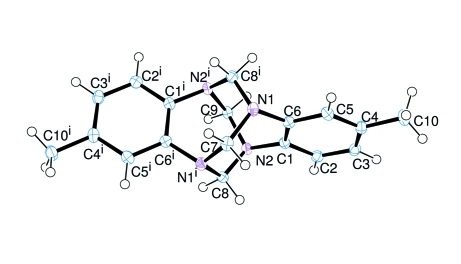
A view of the title molecule with the displacement ellipsoids shown at the 50% probability level with the atomic labelling scheme. The symmetry-related atoms by a crystallographic two-fold axis are indicated by "i".

**Fig. 2.**
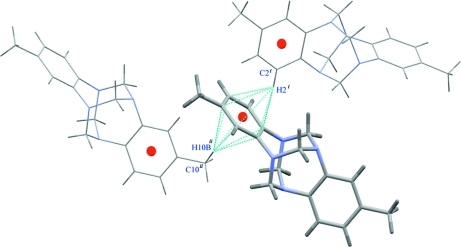
C—H···π-electron arene intermolecular weak contacts in the title structure. The ring centroids are also depicted as red circles. [Symmetry code: (i) 3/2-x,y,1/2+z; (ii) 2-x,1/2-y,-1/2+z.]

## supplementary crystallographic information

### Comment

For many years, cyclic aminals (*gem*-diamine) have attracted intense attention because of their intriguing molecular structures, many of which have been determined by the X-ray crystallography (Murray-Rust, 1974; Murray-Rust & Ridell, 1975; Murray-Rust & Smith, 1975; Glister *et al.*, 2005) and/or by ^1^H- and ^13^C-NMR spectroscopy (Kuznetsov *et al.*, 2007). In the course of the research of the reactivity of aminals we have synthesized crystals of the title compound that contains a cyclic aminal, *i. e.* 2,9-dimethyl-6*H*,13*H*-5:12,7:14-dimethanedibenzo [*d,i*][1,3,6,8]tetraazecine.

The title molecule is shown in Fig. 1. The planes through the symmetry-related aromatic rings are almost perpendicular: the interplanar angle through the atoms C1//C2//C3//C4//C5//C6 and its mentioned symmetry-related plane by (2-*x*,-*y*,*z*) is 81.6 (2)°.

There are only weak intermolecular interactions present in the structure, notably C-H···π-electron ring interactions (Tab. 1, Fig. 2).

### Experimental

A solution of 4-methyl-1,2-diaminebenzene (100 mg, 0.82 mmol) in water (8 ml) and methanol (2 ml) was added dropwise at 278 K to 5 ml of 37% aqueous formaldehyde while stirring it. The reaction mixture was removed from the cooling bath and allowed to warm to room temperature while still stirring it. After stirring at room temperature for 1 h the resultant precipitate was filtered off, washed with water, dried in vacuum and recrystallized from 2-propanol to give the title compound with 65% yield. The melting point of the title structure is 465 K. The melting point was determined visually using glass capillary tube with an Electrothermal melting point apparatus, model 9100, accuracy ±0.5 K, manufacturer: Electrothermal Thermo Scientific.

The NMR spectra were acquired at room temperature on a Bruker AMX 400 Advance spectrometer. ^1^H NMR (δ, 399.9 MHz, CDCl_3_): 2.33, 4.34, 6.92, 6.98. ^13^C NMR (δ, 100.0 MHz, CDCl_3_): 21.0, 68.6, 126.1, 126.7, 136.2, 150.3, 153.2. m/*z* (EI): 292.2 (*M*^+^).

### Refinement

All the H atoms were discernible in the difference electron density maps. However, the H atoms were constrained by the riding model approximation: C—H_methyl_=0.96 Å; C—H_aryl_=0.93 Å; *U*_iso_H_methyl_=1.5*U*_eq_C_methyl_; *U*_iso_H_aryl_=1.2*U*_eq_C_aryl_. In the absence of significant anomalous scattering effects 667 Friedel pairs have been merged.

### Crystal data

**Table:**

C_18_H_20_N_4_	*D*_x_ = 1.343 Mg m^−^^3^
*M**_r_* = 292.38	Melting point: 465 K
Orthorhombic, *A**b**a*2	Mo *K*α radiation, λ = 0.71073 Å
Hall symbol: A 2 -2ac	Cell parameters from 3533 reflections
*a* = 9.9777 (3) Å	θ = 3.0–26.1°
*b* = 18.8351 (4) Å	µ = 0.08 mm^−^^1^
*c* = 7.6963 (2) Å	*T* = 100 K
*V* = 1446.37 (7) Å^3^	Prism, colourless
*Z* = 4	0.16 × 0.15 × 0.06 mm
*F*(000) = 624	

### Data collection

**Table:**

Bruker APEXII CCD diffractometer	737 reflections with *I* > 2σ(*I*)
Radiation source: fine-focus sealed tube	*R*_int_ = 0.041
ω and φ scans	θ_max_ = 26.4°, θ_min_ = 2.2°
Absorption correction: multi-scan (*SADABS*; Sheldrick, 2006)	*h* = −12→12
*T*_min_ = 0.872, *T*_max_ = 0.995	*k* = −23→23
10245 measured reflections	*l* = −9→9
807 independent reflections	

### Refinement

**Table:**

Refinement on *F*^2^	Primary atom site location: structure-invariant direct methods
Least-squares matrix: full	Secondary atom site location: difference Fourier map
*R*[*F*^2^ > 2σ(*F*^2^)] = 0.033	Hydrogen site location: difference Fourier map
*wR*(*F*^2^) = 0.090	H-atom parameters constrained
*S* = 1.06	*w* = 1/[σ^2^(*F*_o_^2^) + (0.0593*P*)^2^ + 0.5089*P*] where *P* = (*F*_o_^2^ + 2*F*_c_^2^)/3
807 reflections	(Δ/σ)_max_ < 0.001
102 parameters	Δρ_max_ = 0.18 e Å^−^^3^
1 restraint	Δρ_min_ = −0.18 e Å^−^^3^
47 constraints	

### Special details

**Table:**

Experimental. The temperature was set with accuracy ±2 K.
Geometry. All s.u.'s (except the s.u. in the dihedral angle between two l.s. planes) are estimated using the full covariance matrix. The cell s.u.'s are taken into account individually in the estimation of s.u.'s in distances, angles and torsion angles; correlations between s.u.'s in cell parameters are only used when they are defined by crystal symmetry. An approximate (isotropic) treatment of cell s.u.'s is used for estimating s.u.'s involving l.s. planes.
Refinement. Refinement of *F*^2^ against ALL reflections. The weighted *R*-factor *wR* and goodness of fit *S* are based on *F*^2^, conventional *R*-factors *R* are based on *F*, with *F* set to zero for negative *F*^2^. The threshold expression of *F*^2^ > 2σ(*F*^2^) is used only for calculating *R*-factors(gt) *etc*. and is not relevant to the choice of reflections for refinement. *R*-factors based on *F*^2^ are statistically about twice as large as those based on *F*, and *R*- factors based on ALL data will be even larger.

### Fractional atomic coordinates and isotropic or equivalent isotropic displacement parameters (Å^2^)

**Table:**

	*x*	*y*	*z*	*U*_iso_*/*U*_eq_	Occ. (<1)
C1	0.8848 (2)	0.09201 (10)	0.1159 (3)	0.0154 (5)	
C2	0.7950 (2)	0.14289 (11)	0.1743 (3)	0.0179 (5)	
H2	0.7273	0.1303	0.2555	0.021*	
C3	0.8041 (2)	0.21256 (11)	0.1138 (3)	0.0189 (5)	
H3	0.7407	0.2469	0.1516	0.023*	
C4	0.9044 (2)	0.23234 (10)	−0.0007 (3)	0.0179 (5)	
C5	0.9937 (2)	0.18036 (11)	−0.0609 (3)	0.0183 (5)	
H5	1.0628	0.1932	−0.1398	0.022*	
C6	0.9825 (2)	0.11024 (11)	−0.0067 (3)	0.0161 (5)	
C7	1.0000	0.0000	−0.1714 (4)	0.0185 (7)	
H7A	0.9337	0.0235	−0.2479	0.022*	0.50
H7B	1.0663	−0.0235	−0.2479	0.022*	0.50
C8	0.8318 (2)	−0.03101 (10)	0.0540 (3)	0.0173 (5)	
H8A	0.7966	−0.0728	0.1173	0.021*	
H8B	0.7556	−0.0095	−0.0093	0.021*	
C9	1.0000	0.0000	0.2796 (4)	0.0157 (7)	
H9A	0.9756	−0.0402	0.3562	0.019*	0.50
H9B	1.0244	0.0402	0.3562	0.019*	0.50
C10	0.9187 (2)	0.30907 (11)	−0.0569 (3)	0.0229 (5)	
H10A	0.8308	0.3322	−0.0538	0.034*	
H10B	0.9545	0.3109	−0.1754	0.034*	
H10C	0.9800	0.3337	0.0221	0.034*	
N1	1.07122 (18)	0.05643 (9)	−0.0750 (2)	0.0177 (4)	
N2	0.87874 (17)	0.02065 (8)	0.1833 (2)	0.0155 (4)	

### Atomic displacement parameters (Å^2^)

**Table:**

	*U*^11^	*U*^22^	*U*^33^	*U*^12^	*U*^13^	*U*^23^
C1	0.0184 (10)	0.0147 (9)	0.0131 (10)	−0.0011 (7)	−0.0031 (9)	0.0001 (9)
C2	0.0181 (10)	0.0192 (10)	0.0163 (11)	−0.0007 (8)	−0.0019 (9)	−0.0009 (9)
C3	0.0212 (10)	0.0174 (9)	0.0179 (11)	0.0028 (8)	−0.0042 (9)	−0.0003 (9)
C4	0.0235 (11)	0.0157 (10)	0.0146 (10)	0.0019 (8)	−0.0050 (10)	−0.0007 (8)
C5	0.0228 (11)	0.0176 (9)	0.0145 (10)	−0.0032 (8)	−0.0006 (9)	0.0008 (9)
C6	0.0177 (10)	0.0171 (10)	0.0136 (10)	−0.0003 (8)	−0.0008 (9)	0.0003 (8)
C7	0.0250 (18)	0.0191 (15)	0.0115 (16)	0.0031 (13)	0.000	0.000
C8	0.0171 (10)	0.0164 (9)	0.0185 (11)	−0.0012 (8)	−0.0021 (9)	−0.0029 (9)
C9	0.0235 (18)	0.0126 (14)	0.0110 (17)	−0.0001 (12)	0.000	0.000
C10	0.0295 (12)	0.0169 (10)	0.0223 (12)	0.0004 (9)	0.0031 (10)	0.0023 (9)
N1	0.0213 (9)	0.0153 (8)	0.0166 (10)	0.0011 (7)	0.0018 (8)	0.0010 (7)
N2	0.0192 (9)	0.0127 (8)	0.0147 (9)	0.0002 (7)	−0.0005 (8)	0.0001 (7)

### Geometric parameters (Å, °)

**Table:**

C1—C2	1.386 (3)	C7—H7A	0.9900
C1—C6	1.399 (3)	C7—H7B	0.9900
C1—N2	1.442 (3)	C8—N1^i^	1.467 (3)
C2—C3	1.395 (3)	C8—N2	1.468 (3)
C2—H2	0.9500	C8—H8A	0.9900
C3—C4	1.385 (3)	C8—H8B	0.9900
C3—H3	0.9500	C9—N2	1.471 (2)
C4—C5	1.403 (3)	C9—N2^i^	1.471 (2)
C4—C10	1.515 (3)	C9—H9A	0.9900
C5—C6	1.390 (3)	C9—H9B	0.9900
C5—H5	0.9500	C10—H10A	0.9800
C6—N1	1.445 (3)	C10—H10B	0.9800
C7—N1^i^	1.478 (2)	C10—H10C	0.9800
C7—N1	1.478 (2)	N1—C8^i^	1.467 (3)
			
C2—C1—C6	119.92 (19)	N1^i^—C8—N2	117.66 (16)
C2—C1—N2	120.06 (19)	N1^i^—C8—H8A	107.9
C6—C1—N2	120.01 (18)	N2—C8—H8A	107.9
C1—C2—C3	120.0 (2)	N1^i^—C8—H8B	107.9
C1—C2—H2	120.0	N2—C8—H8B	107.9
C3—C2—H2	120.0	H8A—C8—H8B	107.2
C4—C3—C2	120.8 (2)	N2—C9—N2^i^	119.5 (3)
C4—C3—H3	119.6	N2—C9—H9A	107.4
C2—C3—H3	119.6	N2^i^—C9—H9A	107.4
C3—C4—C5	118.80 (19)	N2—C9—H9B	107.4
C3—C4—C10	120.40 (19)	N2^i^—C9—H9B	107.4
C5—C4—C10	120.8 (2)	H9A—C9—H9B	107.0
C6—C5—C4	120.8 (2)	C4—C10—H10A	109.5
C6—C5—H5	119.6	C4—C10—H10B	109.5
C4—C5—H5	119.6	H10A—C10—H10B	109.5
C5—C6—C1	119.49 (19)	C4—C10—H10C	109.5
C5—C6—N1	120.5 (2)	H10A—C10—H10C	109.5
C1—C6—N1	120.00 (18)	H10B—C10—H10C	109.5
N1^i^—C7—N1	119.8 (3)	C6—N1—C8^i^	112.77 (17)
N1^i^—C7—H7A	107.4	C6—N1—C7	113.10 (15)
N1—C7—H7A	107.4	C8^i^—N1—C7	114.98 (15)
N1^i^—C7—H7B	107.4	C1—N2—C8	112.79 (17)
N1—C7—H7B	107.4	C1—N2—C9	113.17 (14)
H7A—C7—H7B	106.9	C8—N2—C9	115.38 (15)
			
C6—C1—C2—C3	−1.4 (3)	C1—C6—N1—C8^i^	−70.2 (2)
N2—C1—C2—C3	177.63 (19)	C5—C6—N1—C7	−118.0 (2)
C1—C2—C3—C4	−1.9 (3)	C1—C6—N1—C7	62.4 (3)
C2—C3—C4—C5	2.7 (3)	N1^i^—C7—N1—C6	−77.89 (15)
C2—C3—C4—C10	−176.2 (2)	N1^i^—C7—N1—C8^i^	53.62 (14)
C3—C4—C5—C6	−0.2 (3)	C2—C1—N2—C8	110.4 (2)
C10—C4—C5—C6	178.7 (2)	C6—C1—N2—C8	−70.6 (2)
C4—C5—C6—C1	−3.1 (3)	C2—C1—N2—C9	−116.4 (2)
C4—C5—C6—N1	177.34 (19)	C6—C1—N2—C9	62.7 (3)
C2—C1—C6—C5	3.9 (3)	N1^i^—C8—N2—C1	79.7 (2)
N2—C1—C6—C5	−175.2 (2)	N1^i^—C8—N2—C9	−52.5 (3)
C2—C1—C6—N1	−176.53 (19)	N2^i^—C9—N2—C1	−78.27 (15)
N2—C1—C6—N1	4.4 (3)	N2^i^—C9—N2—C8	53.75 (14)
C5—C6—N1—C8^i^	109.4 (2)		

Symmetry codes: (i) −*x*+2, −*y*, *z*.

### Table 1 Geometry of C—H··· *Cg* interactions (Å,°).

*Cg* denotes the centroid of the benzene ring (C1//C2//C3//C4//C5//C6).

**Table:**

Contact	C–H	C···*Cg*	H···*Cg*	C—H···*Cg*
C2–H2···*Cg*^i^	0.95	3.509 (2)	2.68	147
C10–H10B···*Cg*^ii^	0.98	3.559 (2)	2.61	163

Symmetry codes: (i) 3/2-*x*,*y*,1/2+*z*; (ii) 2-*x*,1/2-*y*,-1/2+*z*.
